# Inhibition of Diverse DsbA Enzymes in Multi-DsbA Encoding Pathogens

**DOI:** 10.1089/ars.2017.7104

**Published:** 2018-09-01

**Authors:** Makrina Totsika, Dimitrios Vagenas, Jason J. Paxman, Geqing Wang, Rabeb Dhouib, Pooja Sharma, Jennifer L Martin, Martin J. Scanlon, Begoña Heras

**Affiliations:** ^1^ Institute of Health and Biomedical Innovation, School of Biomedical Sciences, Queensland University of Technology, Queensland, Australia.; ^2^ Department of Biochemistry and Genetics, La Trobe Institute for Molecular Science, La Trobe University, Bundoora, Australia.; ^3^ Medicinal Chemistry, Monash Institute of Pharmaceutical Sciences, Monash University, Parkville, Australia.; ^4^ Institute for Molecular Bioscience, University of Queensland, Queensland, Australia.

**Keywords:** infection, therapeutics, protein folding, thiol

## Abstract

***Aims:*** DsbA catalyzes disulfide bond formation in secreted and outer membrane proteins in bacteria. In pathogens, DsbA is a major facilitator of virulence constituting a target for antivirulence antimicrobial development. However, many pathogens encode multiple and diverse DsbA enzymes for virulence factor folding during infection. The aim of this study was to determine whether our recently identified inhibitors of *Escherichia coli* K-12 DsbA can inhibit the diverse DsbA enzymes found in two important human pathogens and attenuate their virulence.

***Results:*** DsbA inhibitors from two chemical classes (phenylthiophene and phenoxyphenyl derivatives) inhibited the virulence of uropathogenic *E. coli* and *Salmonella enterica* serovar Typhimurium, encoding two and three diverse DsbA homologues, respectively. Inhibitors blocked the virulence of *dsbA* null mutants complemented with structurally diverse DsbL and SrgA, suggesting that they were not selective for prototypical DsbA. Structural characterization of DsbA-inhibitor complexes showed that compounds from each class bind in a similar region of the hydrophobic groove adjacent to the Cys30-Pro31-His32-Cys33 (CPHC) active site. Modeling of DsbL- and SrgA-inhibitor interactions showed that these accessory enzymes could accommodate the inhibitors in their different hydrophobic grooves, supporting our *in vivo* findings. Further, we identified highly conserved residues surrounding the active site for 20 diverse bacterial DsbA enzymes, which could be exploited in developing inhibitors with a broad spectrum of activity.

***Innovation and Conclusion:*** We have developed tools to analyze the specificity of DsbA inhibitors in bacterial pathogens encoding multiple DsbA enzymes. This work demonstrates that DsbA inhibitors can be developed to target diverse homologues found in bacteria. *Antioxid. Redox Signal.* 29, 653–666.

## Introduction

“Antibiotic resistance is happening right now, across the world” (1). Many common infections that until recently were readily treatable are becoming resistant to most, if not all available, antibiotics, heralding the beginning of a post-antibiotic era, in which common infections will become untreatable and once again lethal. In the era of bacterial multi-drug resistance, many promising new approaches are being investigated to combat these infections. One promising antibacterial strategy is to develop compounds that inhibit bacterial virulence rather than bacterial viability or growth (10, 11, 76). Enzymes and systems required for the biogenesis of virulence factors, the bacterial weaponry necessary for infection, represent attractive target candidates for inhibition as they have the capacity to inhibit a pathogen's ability to cause infection without necessarily affecting its growth or viability, thus reducing selection pressure in bacteria and subsequent development and spread of resistance (28, 67).

Innovation Disulfide bond (Dsb) enzymes are an essential part of the bacterial virulence factor assembly line. Their central role in bacterial pathogenesis makes Dsb oxidoreductases attractive targets for pharmacological intervention, and several drug discovery and development campaigns are currently underway for Dsb inhibitors as novel antibacterial agents. Many pathogens, however, encode multiple diverse DsbA homologues. Here, we studied DsbA inhibition in relevant pathogen*-*specific backgrounds and showed that low-affinity inhibitors can block diverse DsbA enzymes present in clinically important pathogens. This work could guide the future optimization of DsbA inhibitors, rationalization of pathogen-specific DsbA inhibitors, and elaboration of broad-spectrum DsbA antimicrobial leads.

**FIG. 1.**
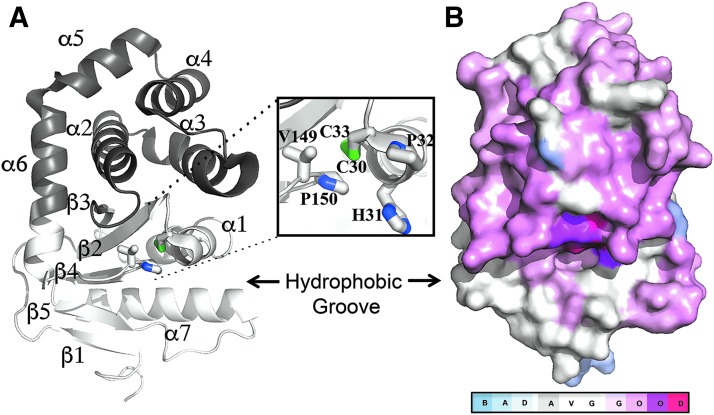
***Escherichia coli*** **DsbA structure and sequence conservation. (A)** Ribbon diagram of EcDsbA [PDB 1FVK (24)]. The TRX-fold and inserted helical domain are shown in *light* and *dark gray*, respectively. Secondary structural features are labeled, and the catalytic disulfide bond is shown in *green*. *Inlet* shows a close-up view of the active site of EcDsbA encompassing a Cys30-Pro31-His32-Cys33 redox-active motif and the adjacent *cis*-proline loop (V149-*cis*Pro150). **(B)** Amino acid sequence conservation in diverse bacterial DsbA prototypes. The conservation scores obtained from an Expresso T-Coffee multiple sequence alignment of 20 DsbA protein sequences are depicted on the surface of EcDsbA by using a color gradient, from *blue* (poorly conserved), through to *white* (reliable conservation) and *red* (well conserved). EcDsbA, *Escherichia coli* disulfide bond; TRX, thioredoxin.

In Gram-negative pathogens, DsbA fulfills these criteria as it works as a folding catalyst for multiple classes of virulence factors but is not necessary for bacterial viability (29). In such pathogens, DsbA is a periplasmic enzyme that catalyzes disulfide bond formation in secreted and outer membrane proteins. As many of these proteins serve virulence functions during infection, DsbA proteins are major facilitators of bacterial pathogenesis (29, 42, 74) and, consequently, DsbA is being targeted for the development of antivirulence drugs (64). Currently, the DsbA oxidative system of *Escherichia coli* K-12 is the best-studied example of oxidative protein folding in bacteria. This pathway involves two enzymes: DsbA, which binds to unfolded polypeptides and oxidizes them *via* a disulfide exchange reaction, and DsbB, which restores the oxidizing activity of DsbA, allowing folding of subsequent substrates to occur. *E. coli* DsbA (EcDsbA) is a thioredoxin (TRX)-like thiol oxidase comprising the characteristic Cys30-Pro31-His32-Cys33 (CPHC) redox active site flanked by a hydrophobic groove required for binding the cognate oxidase EcDsbB (24, 46) (Fig. 1A). Not surprisingly, this groove is the binding site for most EcDsbA inhibitors described to date (4, 18).

Although inhibitor development has focused on the prototypical *E. coli* K-12 DsbA, the increasing number of publicly available sequenced bacterial genomes has revealed that DsbA enzymes are diverse among bacteria (19, 29, 62). These proteins show variable conservation in amino acid sequence and variation in copy number among different bacterial genera, species, or even strains within the same species. For example, uropathogenic *E. coli* (UPEC) strains such as the pyelonephritis isolate CFT073 contain two oxidative systems: the DsbA/DsbB pair, as well as an additional DsbL/DsbI redox pair, which may be dedicated to a specific group of virulence substrates and is lacking from commensal or enteric *E. coli* pathotypes (23, 68). Similarly, certain *Salmonella enterica* serovars (*e.g.*, *S.* Typhimurium) contain an extended number of Dsb proteins, including DsbA/DsbB, DsbL/DsbI, and another virulence plasmid-encoded DsbA homologue called SrgA (9, 30). Multiple copies of DsbA have also been described in other important pathogens such as *Neisseria meningitidis* (63)*, Neisseria gonorrhoeae* (29), and *Pseudomonas aeruginosa* (7, 61), among others.

With such diversity, DsbA presents a unique opportunity as an antivirulence target to customize the specificity of inhibitors to individual pathogens (narrow spectrum) or groups of pathogens sharing a common target (broader spectrum); however, no study to date has investigated DsbA inhibitor specificity in pathogens with multiple diverse DsbA homologues.

The current work investigates the activity of EcDsbA inhibitors, representing two different chemical classes, against two important human pathogens containing multiple diverse DsbA enzymes. We examine the target and pathogen specificity, and we compare at atomic resolution the binding mode of these inhibitors with the diverse DsbAs. Our work provides evidence that low-affinity inhibitors can block diverse DsbA enzymes found in different bacteria or within the same pathogen, and that the extent of inhibition achieved is dependent on the full DsbA complement that each pathogen possesses. Further, this study also highlights conserved residues neighboring the active site of DsbA enzymes that may be exploited in broad-spectrum inhibitor development.

## Results

### Diversity among bacterial DsbA enzymes

DsbA enzymes are found in all classes of Proteobacteria and Chlamydiales as well as in many Fusobacteria and Actinobacteria species (29, 50). A previously reported comparative analysis of 13 structurally characterized DsbA proteins revealed that they form two main structural classes DsbA-I and DsbA-II, which primarily differ on their central β-sheet topology in the TRX-fold, which could be further divided into four subclasses (50). Currently, there are 22 DsbA homologues that have been characterized both structurally and functionally (Supplementary Fig. S1; Supplementary Data are available online at www.liebertpub.com/ars). These are found in representatives of diverse bacterial classes (Actinobacteria, Bacilli, Alpha-, Beta-, and Gammaproteobacteria) and mostly share an overall low identity over their full-length amino acid sequence (between 10% and 56% identity by pair-wise sequence alignment analysis, Supplementary Fig. S1).

The variability at the amino acid level shown by these enzymes prompted us to combine sequence and phylogenetic analyses together with the available structural information to further define the diversity in this family of thiol oxidases. Twenty of the 22 characterized DsbA homologues constituted the group of structurally and functionally defined DsbA proteins used throughout the sequence comparison studies performed hereafter (defined as prototypes). We excluded (i) DsbA from *S. enterica* serovar Typhimurium (SeDsbA) as it is 85% identical to EcDsbA, and (ii) DsbL from UPEC that is 93% identical to its *S. enterica* positional orthologue SeDsbL (30).

We first examined the relationship of the 20 DsbA prototypes at the protein sequence level by constructing a structural multiple sequence alignment (MSA) using the Expresso T-Coffee algorithm. Despite the variable sequence identity (S.I.) in DsbA proteins, the two catalytic cysteines and the *cis*-proline in the active motif are 100% conserved among our set (Fig. 1B). Notably, the hydrophobic binding groove is present within all proteins that are adjacent to the redox active site. There were a number of conserved regions, including the amino acids spanning the catalytic cysteines (Cys30-Pro31-His32-Cys33 in EcDsbA) and the residues preceding the *cis*-proline (Gly149-Val150-*cis*Pro151 in EcDsbA). Other highly conserved hydrophobic residues (Val22, Phe25, Phe26, Leu92, Phe93, and Val155 in EcDsbA) form the core of the DsbA fold and this conservation is probably required for structural stability. Above the groove, the C-terminus of helix α*6* and residues neighboring the catalytic cysteines were moderately conserved. Surprisingly, residues at the C-terminus of helix α*1* and the β5-α*7* region in the TRX domain, which map to the center of the groove, were poorly conserved across DsbA homologues, except for an aromatic residue (Phe36 in EcDsbA) that was found in 16 out of the 20 DsbA prototype structures.

### Phylogenetic analysis assigns bacterial DsbA proteins to three main clades

**FIG. 2.**
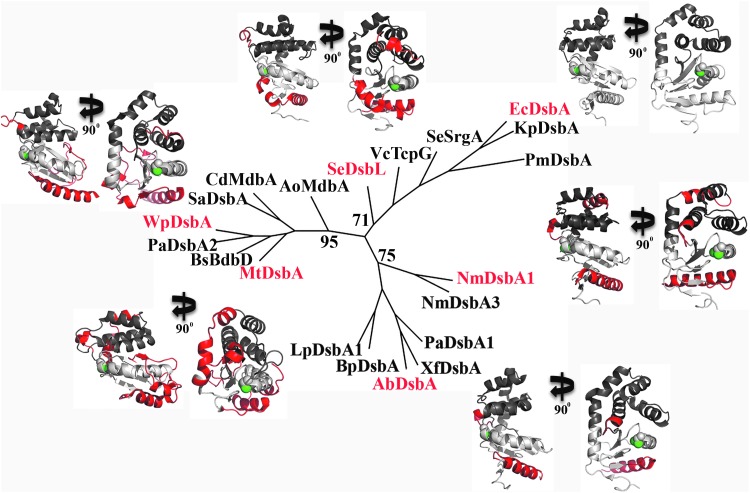
**Diversity in DsbA sequence and structure**. Unrooted Neighbor-Joining consensus tree of 20 DsbA proteins encoded in 17 bacterial species. The tree was based on 1000 bootstrap replicates reconstructed by using protdist and neighbor, the protein distance-based method implemented by PHYLIP (21), using a multiple sequence alignment generated with the Expresso algorithm of the T-Coffee package (66). Proteins are grouped into three well-supported clades (% bootstrap values are shown) and structures from clade representatives (names in *red* font) are shown, highlighting the active site (shown in *green*) and the main structural differences from EcDsbA (shown in *red*). These differences primarily localize in the β5-loop-α*7* region, which for more proteins is truncated and results in a smaller hydrophobic groove. In addition, all proteins clustering with WpDsbA have their β1 strand interacting with β3 (opposite site of the protein) rather than β5. Protein accession numbers and PDB codes are listed in Supplementary Figure S1.

A consensus Neighbor-Joining tree of the 20 DsbA prototypes showed that they mostly grouped into clades that reflected the taxonomic group of their encoding organism, with some exceptions (Fig. 2 and Supplementary Fig. S1). The sequence and structural conservation is clearly apparent for proteins that cluster with EcDsbA [previously defined as DsbA-Ia, (50)], which include homologues from other Gammaproteobacteria such as *Proteus mirabilis* DsbA (PmDsbA, 85% SI and r.m.s.d of 1.1 Å), *Klebsiella pneumoniae* DsbA (KpDsbA, 56% S.I. and r.m.s.d of 0.8 Å), and SeSrgA (35% S.I. and r.m.s.d of 1.4 Å) (30, 33 38, 39). All of these proteins are characterized by long hydrophobic grooves flanking the active site, and they mostly share the same active site residues (Cys-Pro-His-Cys) (30).

A second clade (previously defined as DsbA-Ib) included DsbA proteins from Betaproteobacteria *Burkholderia pseudomallei* (BpDsbA, 27% S.I. and r.m.s.d of 2.1 Å) and *Neisseria meningitidis* (NmDsbA1, 23% S.I. and r.m.s.d of 2.2 Å; NmDsbA3, 22% S.I. and r.m.s.d of 2.2 Å) (35, 41, 71, 72). This clade also contains homologues from Gammaproteobacteria *Xylella fastidiosa* (XfDsbA, 19% S.I. and r.m.s.d of 2.1 Å), *Legionella pneumophila* (LpDsbA1, 25% S.I. and r.m.s.d of 2.3 Å), and one of the DsbA homologues of *Pseudomonas aeruginosa* (PaDsbA1, 30% S.I. and r.m.s.d of 2.2 Å) (37, 56, 60, 61).

The homologues most divergent to EcDsbA (in both sequence and structure) formed a third more taxonomically diverse clade (Fig. 2). These included: *Actinomyces oris* MdbA (AoMdbA, 18% S.I. and r.m.s.d of 3.3 Å), *Mycobacterium tuberculosis* DsbA (MtDsbA, 19% S.I. and r.m.s.d of 2.8 Å), and *Corynebacterium diptheriae* MdbA (CdMdbA, 16% S.I. and r.m.s.d of 2.4 Å) from Actinobacteria; *Bacillus subtilis* BdbD (BsBdbD, 15% S.I. and r.m.s.d of 2.7 Å) and *Staphylococcus aureus* DsbA (SaDsbA, 16% S.I. and r.m.s.d of 1.3 Å) from Gram-positive Firmicutes; *Wolbachia pipientis* DsbA from Alphaproteobacteria (WpDsbA, 10% S.I. and r.m.s.d of 2.9 Å); and the recently described second DsbA homologue of *Pseudomonas aeruginosa* from Gammaproteobacteria (PaDsbA2, 12% S.I. and r.m.s.d of 2.8 Å) (7, 15, 16, 27, 40, 55, 57, 58). These proteins display distinct topology to EcDsbA, where strand β1 is hydrogen bonded to the opposite edge of the core β-sheet (β2–β5) in the TRX domain (Fig. 2). Phylogenetic analysis using maximum likelihood produced an identical tree topology supporting that obtained with distance-based methods (data not shown).

Interestingly, most DsbA prototypes displayed structural features that differed from the archetypal *E. coli* enzyme, in particular, a substantially truncated hydrophobic groove that in *E. coli* DsbA interacts with the partner protein DsbB (34) (Fig. 2). This is more striking in DsbL, the accessory DsbA homologue found in UPEC and *S.* Typhimurium, which has a bent α*6* connecting helix and a six-residue truncation in the β5–α7 motif, which together result in a severely reduced groove. DsbL enzymes additionally show a uniquely electropositive surface as compared with other DsbA homologues that are more hydrophobic (30, 62).

Taken together, phylogenetic and structural comparison of 20 DsbA homologues from diverse bacterial species revealed unique idiosyncrasies despite an overall conserved structure. Such sequence and structural features differentiate DsbA proteins into distinct groups, which are often congruent with bacterial taxonomy (Fig. 2 and Supplementary Fig. S1). This information can serve as a critical guide for predicting the spectrum of activity of DsbA inhibitors.

### Activity of *E. coli* K-12 DsbA inhibitors against pathogens with multiple DsbA enzymes

**FIG. 3.**
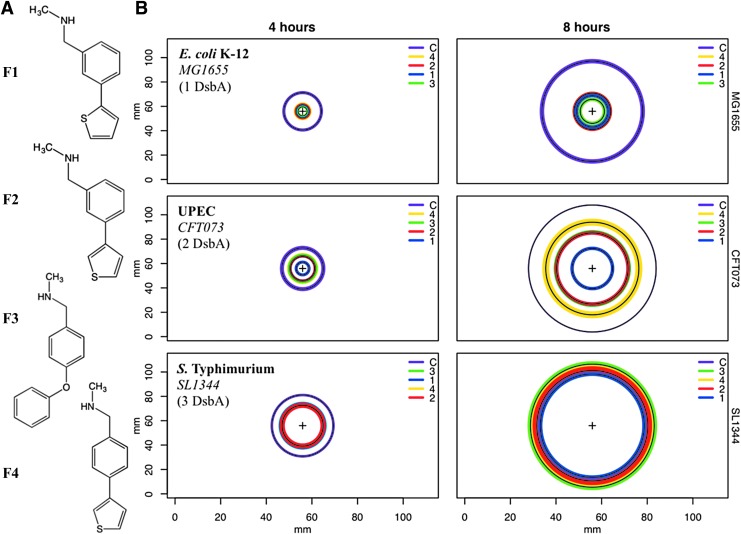
**Pathogen motility inhibition by small-molecule EcDsbA inhibitors. (A)** Chemical structures of phenylthiophene (**F1, F2,** and **F4**) and phenoxyphenyl (**F3**) derivatives previously shown to bind to K-12 EcDsbA (4). **(B)** *Circle plots* of bacterial motility zones (mm) for *E. coli* K-12 MG1655 (*top*), UPEC CFT073 (*middle*), and *S.* Typhimurium SL1344 (*bottom*) cultured for 4 h (*left*) or 8 h (*right*) on soft agar containing inhibitors F1, F2, F3, or F4 at 1 m*M* or 0.1% DMSO (“C,” carrier control). Concentric circles (*black lines*) represent the mean motility diameter (in mm) of *n* = 4 independent replicates, with the standard deviation drawn as a colored zone around each *circle* (*purple* for DMSO control, *blue* for F1, *red* for F2, *green* for F3, and *yellow* for F4). The thickness of the colored zone represents ± one standard deviation value, and the color key is in descending order of the circle diameters graphed in each plot. Mean circle diameters for SL1344 (8 h) on DMSO (*purple*) and F3 (*green*) plates overlap and were the only two groups that were not statistically significant by ANOVA. UPEC, uropathogenic *Escherichia coli*; DMSO, dimethyl sulfoxide.

The high degree of diversity among bacterial DsbA enzymes, in particular among homologues found within the same pathogen, poses the question as to whether our recently developed inhibitors of the archetypal *E. coli* K-12 DsbA (4) will also inhibit diverse DsbA enzymes found in pathogenic *E. coli* strains or other Gram-negative pathogens. To address this, we tested the effect of four small-molecule EcDsbA inhibitors (Fig. 3A) representing two different chemical classes (phenylthiophenes and phenoxyphenyls) on the motility of (i) the reference *E. coli* K-12 strain MG1655 (8) encoding only the prototypical EcDsbA; (ii) the reference UPEC strain CFT073 (51), which encodes DsbA (99.5% identical to K-12 EcDsbA) and EcDsbL (28% identity to EcDsbA); and (iii) the *S.* Typhimurium reference strain SL1344 (31) that encodes SeDsbA (85% identical to EcDsbA), SeDsbL (28% identity to EcDsbA and 93% identity to EcDsbL), and the plasmid-encoded SeSrgA (35% identity to EcDsbA). We have previously shown that, similar to K-12, motility in UPEC CFT073 and *S.* Typhimurium SL1344 can be mediated by the different DsbA homologues found in these pathogens (30, 68), and, therefore, this virulence phenotype can be used as a surrogate method to test the inhibition of DsbA proteins.

The diameter of each strain's motility zone was measured on soft agar containing 1 m*M* of each inhibitor over time (Fig. 3B). As expected, all compounds dramatically inhibited motility in *E. coli* K-12, with MG1655 motility zones being 60–76% smaller on soft agar containing inhibitors than 0.1% dimethyl sulfoxide (DMSO) control plates (Fig. 3B top). Inhibitor impact on UPEC motility was more variable, with relative motility inhibition of CFT073 ranging from ∼70% to 30% with activity following the order: F1>F2>F3>F4 (Fig. 3B middle). For *S.* Typhimurium strain SL1344, with three DsbA homologues, motility inhibition by the four inhibitors was less pronounced, resulting in relative motility zones that were on average 30% smaller than the DMSO control zone (Fig. 3B bottom). By 8 h, SL1344 motility on any inhibitor-supplemented plate only differed from the control plate by an average of <20%. Motility inhibition was not due to inhibition of bacterial growth, as shown for the most active inhibitor F1 at 1 m*M* (Supplementary Fig. S2), strongly suggesting that motility is reduced because of inhibition of DsbA-mediated FlgI folding; however, we cannot exclude that the tested inhibitors may have additional off-target effects that could contribute to the observed phenotype. Taken together, these findings suggest that the four EcDsbA inhibitors are also active against UPEC and *S.* Typhimurium but display a variable activity spectrum, likely due to differences in their specificity for the diverse DsbA enzymes encoded by each of these pathogens.

### Specificity of *E. coli* K-12 DsbA inhibitors against diverse DsbA enzymes

**FIG. 4.**
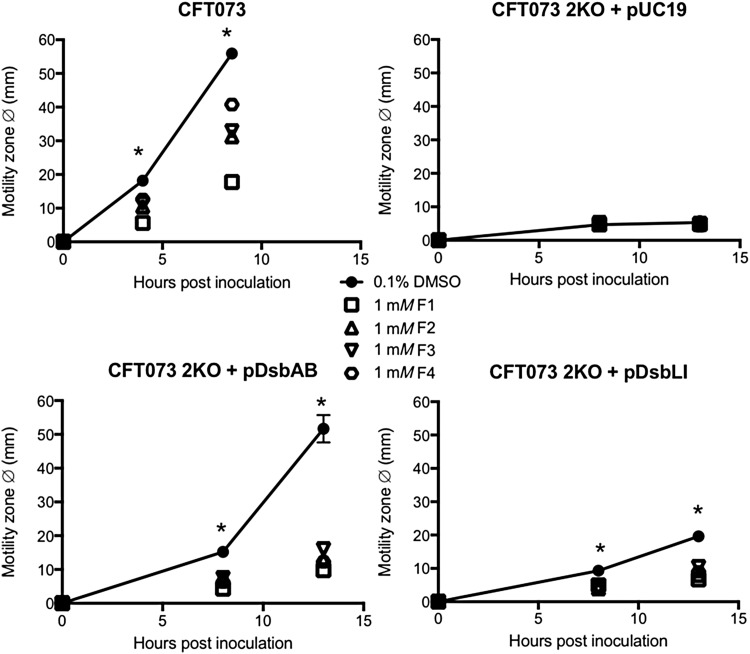
**Specificity of EcDsbA inhibitors for two diverse DsbA homologues in UPEC.** UPEC strain CFT073 encodes DsbA and DsbL, both of which can complement the motility defect of the 2KO mutant when provided *in trans* (2KO + pUC19 empty vector *vs.* 2KO + pDsbAB/pDsbLI), fully or partially, respectively. Restoration of CFT073 2KO motility by either DsbA or DsbL was inhibited by all four inhibitors at a similar level. *Dot plots* represent mean motility zone diameter (mm) ± SEM of four independent replicates for each strain measured on motility plates containing 0.1% DMSO (carrier control; *closed circles* with *line*) or 1 m*M* inhibitor F1, F2, F3, or F4 (open symbols; *squares*, *triangles*, *inverted triangles*, and *hexagons*, respectively), *p < 0.05, ANOVA. SEM, standard error of the mean.

To investigate inhibitor specificity further, we utilized previously constructed and characterized sets of UPEC CFT073 and *S.* Typhimurium SL1344 mutants lacking the full complement of *dsbA* genes (two and three, respectively). The mutants were complemented with each missing homologue on plasmids that were under *lac* operon control (30, 68). Motility assays were conducted as described earlier, except ampicillin and isopropyl β-d-1-thiogalactopyranoside (IPTG) were also included in all soft agar plates to maintain complementation vectors and induce *dsbA* homologue expression. The lack of motility by UPEC mutant CFT073*dsbAB*,*dsbLI* (2KO) was complemented fully by pDsbAB and partially by pDsbLI, as previously reported (68), and this complementation was significantly inhibited by all four inhibitors at 1 m*M* (Fig. 4), suggesting that the inhibitors are active on both of these diverse enzymes.

**FIG. 5.**
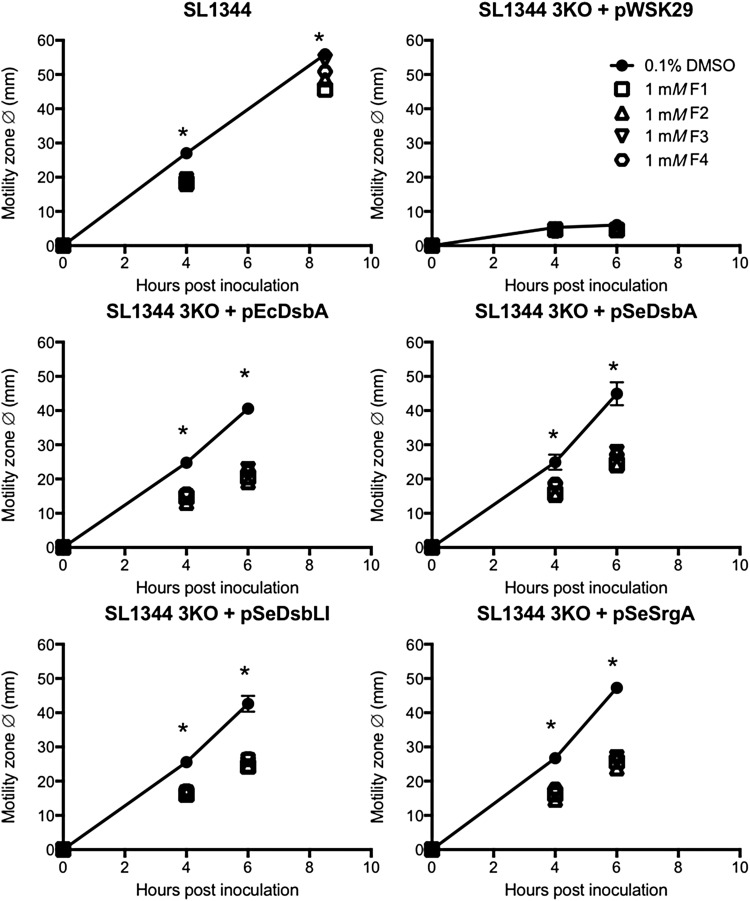
**Specificity of EcDsbA inhibitors for three diverse DsbA homologues in** ***S.*** **Typhimurium.** *S.* Typhimurium strain SL1344 encodes DsbA, DsbL, and SrgA and each homologue can fully complement the motility defect of the 3KO mutant when provided *in trans,* as can the prototypical *E. coli* DsbA (3KO + pWSK29 empty vector *vs.* 3KO + pEcDsbA/pSeDsbA/pSeDsbLI/pSeSrgA). Restoration of SL1344 3KO motility by every homologue was inhibited by all four inhibitors at a similar level. *Dot plots* represent mean motility zone diameter (mm) ± SEM of four independent replicates for each strain measured on motility plates containing 0.1% DMSO (carrier control; *closed circles* with *line*) or 1 m*M* inhibitor F1, F2, F3, or F4 (open symbols; *squares*, *triangles*, *inverted triangles*, and hexagons, respectively), *p < 0.05, ANOVA.

Similarly, the motility defect of the *S.* Typhimurium SL1344*dsbA,dsbLI,srgA* mutant (3KO) was fully complemented by either one of the three native *Salmonella* DsbA homologues (pSeDsbA, pSeDsbLI, pSeSrgA) or the *E. coli* DsbA (pEcDsbA), as previously reported (30), and in all cases, motility restoration was significantly impaired when inhibitors were present in the media at 1 m*M* concentration (Fig. 5). In fact, no differences were seen in the relative motility inhibition obtained with each compound across the different 3KO complemented strains.

**FIG. 6.**
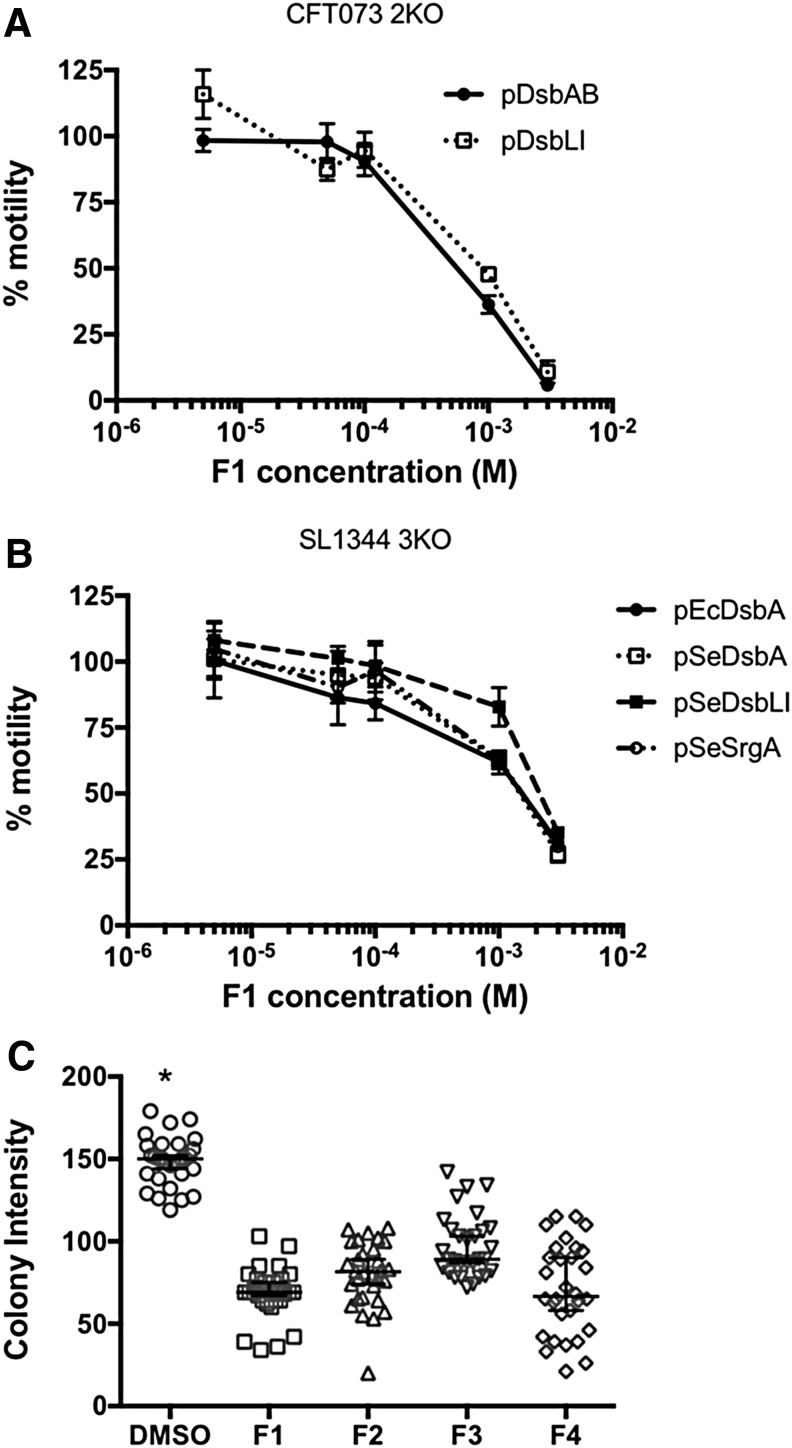
**Dose-dependent inhibition of (A) UPEC and (B)** ***S.*** **Typhimurium motility by compound 1.** Dose-response plots of relative motility for **(A)** UPEC CFT073 2KO transiently expressing DsbAB (*closed circles*) or DsbLI (*open squares*) and **(B)** *S.* Typhimurium SL1344 3KO complemented with EcDsbA (*closed circles*), SeDsbA (*open squares*), SeDsbLI (*closed squares*), or SeSrgA (*open circles*). Relative% motility was calculated by measuring the diameter of the motility zone for each strain in media containing 0.005, 0.05, 0.1, 1, or 3 m*M* of inhibitor F1 and dividing by the diameter of the same strain swimming in DMSO-containing media (carrier control). *Dot plots* represent mean relative motility ± SEM of four independent replicates. **(C)** Inhibition of *S.* Typhimurium DsbL by EcDsbA inhibitors. Intensity of fluorescent colonies of *S.* Typhimurium SL1344 3KO complemented with SeDsbLI. SeDsbLI catalyzes disulfide formation in arysulfate sulfotransferase, which can cleave MUS to release a fluorescent product. Bacteria were cultured in lysogeny broth-MUS agar plates with or without inhibitors (1 m*M*) and imaged in a BioRad GelDoc. The colony intensity of 30 colonies per plate was measured as adjusted volume (background-adjusted sum of all intensities within the colony boundaries) in Image Lab 5.0 and is shown as *dot plots* with group means and 95% confidence intervals. Addition of F1, F2, F3, or F4 in the agar resulted in a significant reduction in mean colony intensity compared with DMSO-containing plates, *p < 0.05, ANOVA. MUS, methylumbelliferyl sulfate; SeDsbA, DsbA from *S. enterica* serovar Typhimurium.

To investigate whether motility inhibition was concentration dependent, inhibitors were further tested in dose-response motility assays by using both sets of UPEC and *S.* Typhimurium mutants and complemented strains. For UPEC, inhibitory effects were only observed at F1 concentrations of 1 m*M* or higher, with no motility inhibition seen at 100, 50, or 5 μ*M*; this was true for both DsbA- and DsbL-mediated UPEC motility (Fig. 6A). For *S.* Typhimurium, motility inhibition by F1 followed a more gradual dose response in all complemented strains (Fig. 6B). Similar inhibitory effects were seen with inhibitors F2, F3, and F4 (Supplementary Fig. S3), suggesting that all compounds can act on the DsbA homologues in a dose-dependent fashion.

The ability of all compounds to inhibit the highly diverse DsbL homologue was further confirmed by monitoring the functional activity of arysulfate sulfotransferase (AssT) in SL1344, a native substrate of DsbL that requires disulfide bond formation for its functional folding and catalytic activity (30). Active AssT cleaves 4-methylumbelliferyl sulfate (MUS) to generate a fluorescent product. When plated on selective media containing IPTG and MUS, the intensity of 3KOpDsbLI fluorescent colonies was significantly decreased when inhibitors were present at 1 m*M*, as compared with the DMSO control (Fig. 6C). Taken together, these findings demonstrate that our EcDsbA inhibitors F1–F4 can interact and inhibit diverse DsbA homologues found in two species of pathogenic bacteria.

### Atomic characterization of EcDsbA–inhibitor complexes

**FIG. 7.**
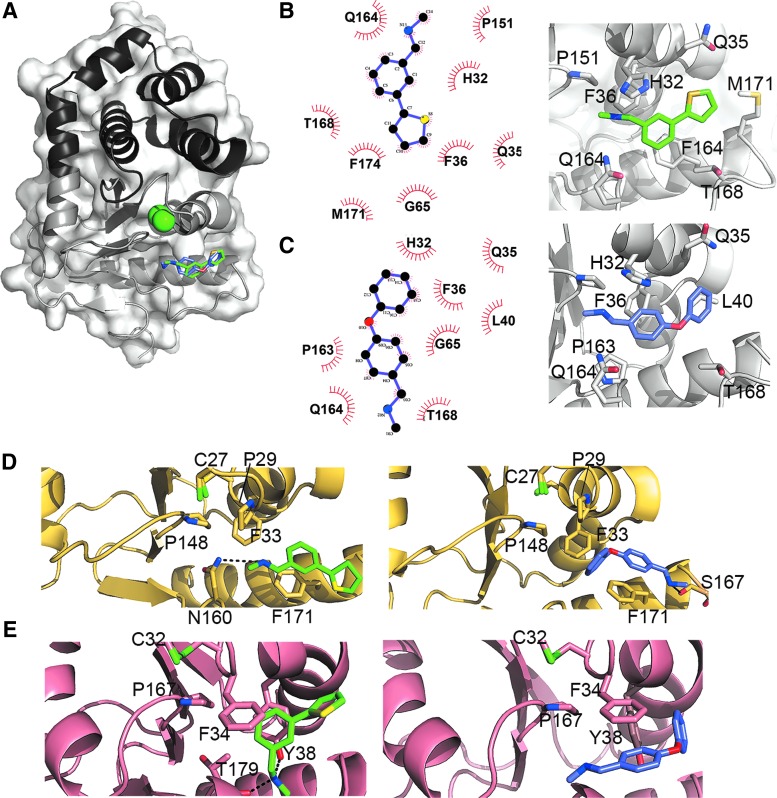
**Structural characterization of EcDsbA in complex with inhibitors F1 and F3 and molecular docking of F1 and F3 with SrgA and DsbL.** **(A)** Superposition of the co-crystal structures of EcDsbA-F1 and EcDsbA-F3 complexes showing that both inhibitors bind to a similar part of the EcDsbA hydrophobic groove. The position of the active site cysteine residues is shown in *green*. **(B, C)** PDBsum analysis (*lig-plot*, *left panel*) and close-up view (*right panel*) of the EcDsbA-F1 and EcDsbA-F3 complexes, respectively. **(D, E)** Binding mode of *top* ranked docked poses of F1 (*green*) and F3 (slate *blue*) into SrgA **(D)** and DsbL **(E)** hydrophobic clefts, obtained by using AutoDock Vina. For clarity, only interacting residues and the active site cysteines and *cis*-proline are displayed in sticks style. F1 and F3 are predicted to be stabilized by Π interactions with aromatic residues in both SrgA (F171) and DsbL (F34 and Y38). In addition, H-bonds can occur between F1 and SrgA N160, F3 and the carbonyl of SrgA S167. Similarly, F1 can form an H-bond with Y38 and the main chain carbonyl of T179 in DsbL. A likely shift in DsbL K37 would allow it to hydrogen bond to the sulfur and oxygen groups of F1 and F3, respectively. Similarly, a possible shift in the sidechain of DsbL Q164 would allow another interaction with F3 nitrogen. All hydrogen bonds are depicted by *black*-*dashed lines*.

To better understand the mode of action of our EcDsbA inhibitors, we structurally characterized one phenylthiophene (F1) and one phenoxyphenyl (F3) derivative in complex with EcDsbA and defined their mode of binding (Fig. 7A). F1 and F3 were individually soaked at 10 m*M* concentration into preformed EcDsbA crystals, and the structures of these complexes were determined at 1.99 Å resolution (Supplementary Table S1). The co-crystal structures revealed that both molecules bind to EcDsbA in a similar region of the hydrophobic groove near the CPHC motif (Fig. 7A–C and Supplementary Fig. S4). To accommodate these compounds in the cleft, the loop linking β5 with α*7* undergoes a substantial conformational change, which has been observed for other EcDsbA inhibitors on binding (4). F1 and F3 are stabilized primarily by hydrophobic contacts with His32, Gln35, Phe36, Gly65, Pro151, Gln164, and Thr168 (Fig. 7B and C). In addition, the phenyl ring of F1 forms a partial stacking with Phe174 and neighbors Met171 (Fig. 7B), whereas F3 flanks residues Leu40 and Pro163 (Fig. 7C). The binding of these small molecules to the hydrophobic groove of EcDsbA would obstruct the interaction with and reoxidation by EcDsbB, resulting in a pronounced reduction of EcDsbA function as shown by the dramatic inhibition of motility observed for *E. coli* K-12 (Fig 3B).

### Molecular docking of EcDsbA inhibitors F1 and F3 with DsbL and SrgA

F1 and F3 resulted in motility inhibition in UPEC and *S.* Typhimurium strains expressing the diverse DsbA homologues, DsbL and SrgA (Figs. 4 and 5). AutoDock Vina was used to explore the molecular docking of F1 and F3 with SeSrgA and SeDsbL. First, we assessed the ability of this molecular docking tool to reproduce the previously identified binding mode of these inhibitors to EcDsbA. In both cases, the top docked conformation out of a total of nine binding conformations, based on predicted affinity (kcal/mol), closely approximated the positioning of the inhibitors in the crystal structures (Supplementary Fig. S5A).

We then docked F1 and F3 into SeSrgA and SeDsbL by using the same parameters as for the control run with EcDsbA, except for the inclusion of some flexible amino acid side chains for the DsbA homologues. Specifically, DsbL Lys37 and Tyr38 were made flexible along with SrgA Asn160 as they partly occluded the hydrophobic cleft in their crystal structures. Docking conformations were ranked on the predicted binding affinities and resemblance to the experimental EcDsbA-F1/F3 binding modes seen in the crystal structures. In all cases, the final conformations selected (Fig. 7D, E and Supplementary Fig. S6) were in the top three out of nine binding modes based on predicted affinities, with the top three binding conformations being largely similar (Supplementary Fig. S5B, C). Resembling the EcDsbA-F1/F3 complexes, the hydrophobic nature of SeSrgA and SeDsbL clefts along with the aromatic nature of the F1 and F3 inhibitors means that Π–Π aromatic contacts are the predominate interactions (Π–Π interactions with Phe171 in SeSrgA and Phe34, Tyr38 in SeDsbL) that stabilize F1 and F3 in the hydrophobic groove of these enzymes. These docking models also show that backbone and side chain-mediated hydrogen bonds can also occur between the inhibitors and the diverse DsbA homologues (Fig. 7D, E, Supplementary Fig. S6).

## Discussion

Inhibition of oxidative protein folding in bacteria is considered a promising antimicrobial approach that could provide urgently needed solutions to the global problem of rising drug resistance and the paucity in new antibiotic development. Most Gram-negative bacteria contain dedicated disulfide bond (Dsb) machinery to efficiently catalyze oxidative disulfide formation, which is an all-important step in the assembly of a wide array of bacterial virulence proteins, from adhesion factors and components of secretory machineries to toxins, flagella, and other critical virulence enzymes (29).

The thiol-oxidizing DsbA/DsbB system is currently the main target for pharmacological inhibition (64) as it constitutes the major player in virulence factor oxidative folding, and deletion of *dsbA/dsbB* genes in numerous pathogens results in significant or total reduction in virulence [reviewed in Refs. (29) and (62)]. Bacteria, however, display considerable diversity in their disulfide folding enzymes (17, 25, 59), particularly among DsbA thiol oxidases (29, 36, 50), raising questions about the spectrum of activity that can be achieved by some of the already developed inhibitors. Here, we utilized a combinatorial approach to explore the specificity of two classes of recently developed *E. coli* DsbA inhibitors against diverse DsbA targets found in two important human pathogens.

UPEC and *S.* Typhimurium are ideal organisms for exploring the therapeutic application of DsbA inhibitors. First, they are two of the top World Health Organization (WHO) listed pathogens of international concern due to their high antibiotic resistance rates and a combined global burden of more than 200 million infections annually (1). UPEC are the leading cause of urinary tract infections (UTIs) in hospitals and the community and a frequent cause of bloodstream infections (69). An estimated 150 million UTIs occur globally each year, costing more than 6 billion dollars in direct healthcare expenditure (22). Nontyphoidal *S. enterica* serotypes such as Typhimurium are the main cause of foodborne diarrhea, with an estimated burden of 94 million gastroenteritis cases and 1,55,000 global deaths each year (45).

Resistance to fluoroquinolones (one of the most widely used oral antibiotics) is globally high in *E. coli* and *Salmonella*, with rates exceeding 50% in 5/6 of the WHO regions and 25% in 3/6 WHO regions, respectively (1). Even higher rates of third-generation cephalosporin resistance in *E. coli* (>65% in 6/6 WHO regions), conferred by extended spectrum beta-lactamase enzymes, means that treatment of multidrug resistant (MDR) *E. coli* infections must now rely on last-line drugs, such as carbapenems or colistins. Worryingly, both carbapenem and colistin resistance has already been reported in MDR *E. coli* (44, 49, 54), highlighting the challenge that clinicians now face in treating pan-resistant *E. coli* infections worldwide (3).

UPEC and S. Typhimurium are also ideal pathogens for studying DsbA inhibitor specificity as they represent a large group of human, animal, and plant pathogens that encode not just one but also multiple and diverse DsbA homologues in their genome (29): DsbA and DsbL for UPEC, and DsbA, DsbL, and SrgA for *S.* Typhimurium. These homologues fulfil similar redox roles *in vivo*; however, they show differences with regard to their substrate repertoire in each pathogen (23, 68). SrgA is a close structural homologue of the prototypical *E. coli* K-12 DsbA but bears a different redox active site and redox properties (30). DsbL is a more distant homologue of EcDsbA in both sequence and 3D architecture, with one of the main differences being a truncated hydrophobic groove (23, 30). Given that the groove is the area of the enzyme where most described EcDsbA inhibitors were shown to bind to date [reviewed in Ref. (64)], this would suggest that such inhibitors might display variable activity against DsbL and SrgA. Our data for phenylthiophene and phenoxyphenyl class inhibitors, however, argue against this, as we have clearly evidenced that: (i) these inhibitors can be readily accommodated in the cleft of DsbA, SrgA, and DsbL and (ii) that they can block the function of all enzymes in UPEC and *S.* Typhimurium *in vivo* to an extent that attenuates virulence at similar levels.

Interestingly, when inhibitor impact on motility was assessed on wild-type strains of UPEC and *S.* Typhimurium (each encoding their full complement of DsbA homologues), the extent of motility inhibition was more variable (in the case of UPEC) or less pronounced (in the case of *S.* Typhimurium) than when assessed on complemented mutants encoding each homologue *in trans*. The explanation of a simply higher DsbA copy-number present in wild-type strains *versus* complemented mutants is probably unlikely to fully account for the observed differences, given that all mutants in our study were complemented with high copy-number plasmids, and hence expressed each DsbA homologue at higher levels than the wild-type strains (30, 68). This difference more likely suggests that the copy-number and biology of DsbA homologues found in each pathogen (*i.e.*, enzyme sequence, structure, redox properties, gene expression, and full repertoire of substrates folded) determine the activity of each inhibitor. This highlights the significance of testing DsbA inhibitors directly on wild-type pathogenic isolates with a full complement of native DsbAs, such as those used in this study, instead of laboratory strains or non-pathogenic isolates, and this would also allow for testing whether resistance to such inhibitors could develop by homologue transfer.

Moreover, our work highlights that the abundance of structure-function information on bacterial DsbA proteins can allow insightful predictions to be made on their potential druggability based on their grouping into distinct clades. This would greatly assist the development of antimicrobials with a customized spectrum of activity. Although DsbA, DsbL, and SrgA are diverse homologues (in sequence and structure) found within the same pathogen, when examining their diversity in light of the wider diversity observed in 20 structurally and functionally characterized DsbA enzymes from distant bacterial species, we found that DsbA, SrgA, and DsbL all cluster together in clade 1 and are representative of the structural diversity observed within this clade. This would predict that EcDsbA inhibitors could be active against homologues within this clade, which was supported by our functional, structural, and modeling data. This notion is further supported by recent studies showing that PmDsbA and KpDsbA, which are also members of clade 1, interact and can be blocked by other EcDsbA inhibitors (38, 39), whereas homologues from clade 2 and 3 remain active (unpublished data). Similarly, inhibitors developed against any DsbA representative from clade 2 or 3 could be expected to display activity across organisms encoding other homologues for the same clade, and as clade 3 is highly taxonomically diverse, this would suggest that the development of broad-spectrum DsbA inhibitors may be a possibility.

We have demonstrated that phenylthiophene and phenoxyphenyl inhibitors of EcDsbA are active against DsbL and SrgA, which are distinct homologues encoded together with DsbA in the important human pathogens UPEC and *S.* Typhimurium. Our atomic resolution co-crystal structures revealed a binding mode for these small-molecule inhibitors to EcDsbA that would obstruct its interaction with the cognate oxidase EcDsbB, therefore altering DsbA redox homeostasis and markedly decreasing DsbA function. This mode of binding was closely mimicked by the modeled interactions seen for our inhibitors with the structurally diverse SrgA and DsbL, which would predict a similar inhibition of function to that of EcDsbA and that we confirmed functionally in different bacterial virulence assays.

From the positioning of our inhibitors in the protein structures and the residue conservation in neighboring areas, one can obtain information for the requirements of DsbA inhibitor design to increase inhibitor potency and/or customize the inhibitor activity spectrum. Both phenylthiophene and phenoxyphenyl inhibitors bound to the same region in the groove and were mostly surrounded by aromatic residues. Key amino acids included histidine 32 and phenylalanine 36 and 174 (*E. coli* DsbA numbering), which form hydrophobic interactions with the inhibitors, mainly Π–Π aromatic interactions. Some of these residues were found to be physicochemically conserved across diverse DsbA homologues; histidine 32 is present in 65% of the analyzed prototypes and in the remaining proteins this residue is most commonly aromatic (phenylalanine or tyrosine). Similarly, phenylalanine 36 is 65% conserved and the exceptions have mostly a tyrosine or leucine in this position. A conserved glutamine/asparagine at position 164 (50% conserved across prototypes) brings a polar group to interact with the inhibitors.

Conservation of these important residues across distinct DsbA homologues dictates that hydrophobic compounds that can maintain the conserved interactions would bind and partially block other diverse DsbA homologues. Future efforts for increasing the potency and spectrum of activity of the inhibitors should consider highly conserved residues surrounding the active site of DsbA. Expanding the structure of the inhibitors to interact with the conserved cysteines in the active site and/or the residues in the *cis*-proline loop ([Gly/Ala] [Val/Thr] *cis*Pro) may be a useful approach to develop more potent inhibitors while maintaining a broad spectrum of activity.

## Materials and Methods

### Sequence and phylogenetic analysis of DsbA homologues

We carried out a Dali search (32) by using the structures of EcDsbA [PDB code 1FVK, (24)], SaDsbA [PDB code 3BCI, (27)], and DsbL [PDB code 3C7M (23)] as our query models to identify all DsbA homologues available in the RCSB Protein Data Bank. Given that DsbA-like proteins share low SI, we utilized different methods to curate our list of DsbA homologues and confirm that the identified proteins were, indeed, DsbA homologues. All identified DsbA structures were manually analyzed for the presence of the structural hallmarks of DsbA homologues (inserted alpha helical domain and the TRX-fold encompassing a CXXC motif and a *cis*-Proline loop). Further, structures and corresponding sequences were retained only where published experimental data confirmed that the identified enzymes functioned as thiol oxidases (Supplementary Fig. S1).

The corresponding full-length amino acid sequences of 22 DsbA proteins matching the criteria described earlier were retrieved, and a non-redundant set of 20 (prototypes) was used in subsequent analyses (DsbA from *S. enterica* and DsbL from UPEC were excluded due to high sequence and structural identity to their positional orthologues in *E. coli* and *S. enterica,* respectively; Supplementary Fig. S1). Multiple sequence alignments of the 20 DsbA prototypes were carried out by using three protein alignment methods of the T-Coffee MSA package: (1) Expresso structural alignment (6), (2) PSI-Coffee Homology extension (14), and (3) M-Coffee combining popular aligners (73). Alignment accuracy was evaluated by TCS (12, 13) and by confirming that the two completely conserved features of the DsbA active site (the characteristic CXXC catalytic motif and the *cis*-Proline loop), which are adjacent in three-dimensional space but distant in primary sequence, were aligned among all sequences. The Expresso structural alignment was selected and used to reconstruct an unrooted Neighbor-Joining and maximum likelihood consensus phylogenetic tree based on 1000 bootstrap replicates using the PHYLIP package (21). The accession number and PDB code for each DsbA homologue is shown in Supplementary Figure S1.

### Bacterial strains, plasmids, and culture conditions

All bacterial strains used in this study (Supplementary Table S2) were routinely cultured at 37°C on solid or in liquid lysogeny broth (LB) medium supplemented, where necessary with kanamycin (km, 50 μg mL^−1^) or ampicillin (amp, 100 μg mL^−1^). Culture media were supplemented with 1 μ*M* isopropyl IPTG to induce expression of DsbA, DsbL, and SrgA from plasmids pDsbA, pDsbLI, and pSrgA, respectively. Construction of UPEC and *S.* Typhimurium mutants and plasmids was previously described (30, 68).

### Motility assays

Swimming motility of *E. coli* and *S.* Typhimurium strains was assessed as previously described (30, 68). Briefly, 2 μl of four independent liquid overnight cultures of each strain was inoculated onto the surface of LB semi-solid (0.3% w/v) agar containing DMSO or inhibitors (phenylthiophenes and phenoxyphenyl) at various concentrations (5–3 m*M*). Plates were incubated at 37°C, and the diameter of bacterial outward growth was measured in millimeters at various time-points. The mean motility zone diameter for each strain was calculated from four replicates tested under each condition, and group means were compared by one-way ANOVA (statistical significance set at *p* < 0.05). Circle plots of mean motility zone diameters for wild-type strains (Fig. 3B) were generated in R (2) by using the functions of the package “plotrix” (43). All other data graphs were generated in GraphPad Prism.

### AssT activity assays

AssT enzyme activity was monitored at a colony level by using an agar plate assay, as previously described (30, 75). Briefly, strain SL1344 3KOpSeDsbLI was streaked on LB agar plates containing 0.1 m*M* 4-MUS (Sigma, Castle Hill, Australia) and 0.1% DMSO or 1 m*M* inhibitors F1–F4. The sulfate of the 4-MUS in the medium is cleaved by AssT forming 4-methylumbelliferone, a fluorescent product that can be detected under UV light (320 nm). Plates were incubated at 37°C overnight. Functional AssT production requires DsbL-mediated disulfide-bond formation and in conditions where functional AssT enzyme is produced *S.* Typhimurium colonies fluoresce brightly under UV light. Plates were UV exposed and imaged simultaneously in a BioRad GelDoc. The colony intensity of 30 colonies per plate was measured as adjusted volume (background-adjusted sum of all intensities within the colony boundaries) in Image Lab 5.0. Group means were compared by one-way ANOVA (statistical significance set at *p* < 0.05).

### Expression and purification of EcDsbA

Recombinant EcDsbA was expressed and purified as previously described (4, 53). Briefly, *E. coli* BL21 (DE3) carrying native EcDsbA encoding plasmid were grown for 24 h at 30°C in ZYM-5052 autoinduction media (65) supplemented with 50 μg/mL kanamycin.

Cells were harvested by centrifugation, and the periplasmic fraction was obtained by cold osmotic shock (26). On addition of 0.8 *M* (NH_4_)_2_SO_4_, the periplasmic fraction was loaded onto a HiLoad 1610 Phenyl Sepharose HP column (GE Healthcare) equilibrated in 20 m*M* 2-amino-2-(hydroxymethyl)propane-1,3-diol (TRIS) (pH 8.0), 50 m*M* NaCl, and 1 *M* (NH_4_)_2_SO_4_. The bound proteins were eluted on a gradient from 1–0 *M* (NH_4_)_2_SO_4_. Fractions containing EcDsbA were buffer exchanged into 25 m*M* 4-(2-hydroxyethyl)piperazine-1-ethanesulfonic acid (HEPES) (pH 6.8), and the protein was further purified by anion-exchange chromatography on a Mono Q 5/50 GL column (GE Healthcare). EcDsbA was then oxidized by the addition of 1.7 m*M* copper(II)[1,10-phenanthroline], and the protein was purified to homogeneity by using a HiLoad Superdex S-75 size-exclusion chromatography column (GE Healthcare) equilibrated in 25 m*M* HEPES (pH 6.8), 150 m*M* NaCl. Protein purity was confirmed by sodium dodecyl sulfate-polyacrylamide gel electrophoresis.

### Crystallization and structure determination of EcDsbA-inhibitor co-crystals

Crystals of EcDsbA were grown by the hanging drop vapor-diffusion method using previously established conditions (47). Briefly, 1 μL of 30 mg/mL EcDsbA was mixed with 1 μL of crystallization buffer (11–13% polyethylene glycol [PEG] 8000, 5% glycerol, 1 m*M* CuCl_2_, 100 m*M* sodium cacodylate pH 6.1–6.4) and equilibrated against 0.5 mL of crystallization buffer at 18°C. Typically, large crystals (0.6 × 0.4 × 0.2 mm) were obtained after 2–3 days of incubation.

Crystal soaking was carried out by transferring EcDsbA crystals into 2 μL drops of 24% PEG 8000, 22% glycerol, 100 m*M* sodium cacodylate pH 6.1–6.4 containing compound 1 or compound 3 at a final concentration of 10 m*M* (2–5% of DMSO) and incubating them for 2 h. Crystals were mounted into loops and flash-cooled in liquid nitrogen before data collection. Diffraction data for complexes 1 and 3 were collected at the UQ ROCX facility (using a Rigaku FR-E Superbright X-ray generator and a Rigaku Saturn 944 CCD detector) and the Australian Synchrotron, respectively. Overall, 0.5° or 1° oscillation images were collected for a total of 180*°*. Diffraction data was indexed and integrated with CrystalClear 1.4. (Rigaku) or HKL2000. Phasing was carried out by molecular replacement with Phaser (48) by using the structure of EcDsbA (PDB code 1FVK) as a search model (24). The final models of EcDsbA in complex with compound 1 and 3 were completed by iterative cycles of model building and refinement by using Coot (20) and phenix.refine (5). Data collection and refinement statistics are summarized in Supplementary Table S1. Generation of molecular figures was carried out with PyMOL v1.7.0.5. The structures were submitted to the PDB under the codes 6BR4 and 6BQX.

### Docking methods

The computational molecular docking tool AutoDock Vina (70) was used to predict the binding mode of F1 and F3 to DsbL and SrgA. The protocol was first validated by docking F1 and F3 into EcDsbA and comparing the docking results with the experimental crystal structures of EcDsbA in complex with those inhibitors. The docking protocol involved preparing the pdbqt files of proteins and ligands using AutoDockTools (52). The apo form of EcDsbA was set as a rigid structure, where a 24 × 16 × 24 Å search space was set up to cover the entire hydrophobic cleft. Standard chemical bond torsions were applied to F1 and F3, which were docked to EcDsbA by using AutoDock Vina to calculate binding affinity. For each inhibitor, the results were ranked on the basis of predicted free energy of binding and the conformations that most closely approximated the crystal structures. For docking F1 and F3 to SrgA and DsbL, the x-ray crystallographic structures of these proteins were recovered from the protein data bank (3TRK, and 3N41); any co-crystallized ligand and water molecules were removed; and F1 and F3 were docked to the macromolecules by using AutoDock Vina. The interaction between ligand and macromolecule was visualized by using the PyMOL molecular graphics system (PyMOL v1.7.0.5).

## Supplementary Material

Supplemental data

## Abbreviations Used

ARC: Australian Research Council
AssT: arysulfate sulfotransferase
CPHC: Cys30-Pro31-His32-Cys33
DMSO: dimethyl sulfoxide
Dsb: disulfide bond
EcDsbA: *Escherichia coli* DsbA
HEPES: 4-(2-hydroxyethyl)piperazine-1-ethanesulfonic acid
IPTG: isopropyl β-_D_-1-thiogalactopyranoside
LB: lysogeny broth
MDR: multidrug resistant
MSA: multiple sequence alignment
MUS: methylumbelliferyl sulfate
PEG: polyethylene glycol
SeDsbA: DsbA from *S. enterica* serovar Typhimurium
SEM: standard error of the mean
S.I.: sequence identity
TRX: thioredoxin
UPEC: uropathogenic *Escherichia coli*
UTIs: urinary tract infections
WHO: World Health Organization
