# Supplementary material for: Inhibition of Diverse DsbA Enzymes in Multi-DsbA Encoding Pathogens
Source: Antioxid Redox Signal. 2018 Sep 1;29(7):653–66. doi: 10.1089/ars.2017.7104 (PMC6067686; doi:10.1089/ars.2017.7104)
Supplement: Supplemental data [file Supp_Data.pdf]

Supplementary Data

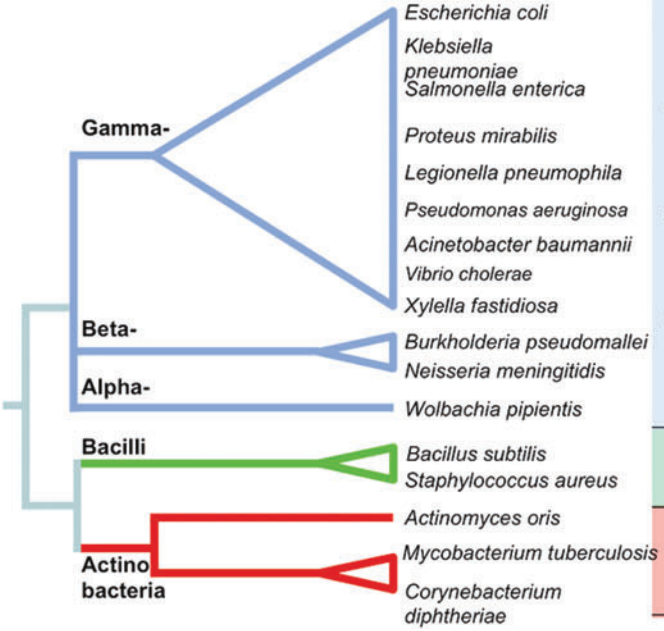

Phylum	Protein	Gene ID	Locus Tag	PDB code	Year of deposition	Resolution Å	% aa sequence ID to EcDsbA	Rmsd (Å, #Cα) to 1FVK	Active sites	XX cis Pro	Ref
Proteobacteria	EcDsbA	948353	b3860	1DSB/1FVK	1993/1996	2.00/1.70	100	N/A	CPHC	GVP	(24)
	EcDsbL	1039926	c3786	3C7M	2008	1.55	24	2.1,162	CPFC	GVP	(23)
	KpDsbA	17434048	KPK_5512	4MCU	2013	1.99	81	0.8, 182	CPHC	GVP	(39)
	SeSrgA	1256229	PSLT011	3L9V	2010	2.15	35	1.4, 172	CPPC	GTP	
	SeDsbA	1255523	STM3997	3L9S	2010	1.58	85	0.7, 178	CPHC	GVP	
	SeDsbL	1254716	STM3193	3L9U	2010	1.57	25	2.1, 161	CPFC	GVP	(30)
	PmDsbA	6800320	PMI2828	4OCE	2014	1.77	56	1.1, 179	CPHC	GVP	(38)
	LpDsbA1	19831690	lpg0123	4JRR	2013	1.88	25	2.3, 159	CPWC	AVP	(37)
	PaDsbA1	877731	PA5489	3H93	2009	1.50	30	2.2, 164	CPHC	GVP	(61)
	PaDsbA2	12576066	PA14_59960	4N30	2013	1.30	12	2.8, 116	CPFC	ATP	(7)
	AbDsbA	6002733	ABAYE3833	4P3Y	2014	2.15	31	2.3, 163	CPHC	GVP	(56)
	VcTcpG	2614468	VC0034	1BED	1996	2.00	40	1.3, 167	CPHC	GVP	(33)
	XfDsbA	1126982	XF1436	2REM	2007	1.90	19	2.1, 167	CPHC	GTP	(60)
	BpDsbA	3093149	BPSL0381	4K2D	2013	1.90	27	2.1, 160	CPHC	GVP	(35)
	NmDsbA1	902389	NMB0278	3DVW/3A3T	2008/2009	1.50/2.10	23	2.2,157	CPHC	GTP	(41,71)
	NmDsbA3	902521	NMB0407	3DVX/2ZNM	2008	2.80/2.30	22	2.2,152	CVHC	STP	(41,72)
	WpDsbA	2738597	WD_1055	3F4R	2008	1.60	10	2.9, 137	CYHC	ATP	(40)
F	BsBdbD	936031	BSU3345	3GH9	2009	1.69	15	2.7, 137	CPSC	ATP	(16)
	SaDsbA	11935157	MW2331	3BCI	2007	1.81	16	1.3, 167	CPYC	TTP	(27)
A	AoMdbA	1043210130	ANA_1994	4Z7X	2015	1.55	18	3.3, 141	CSHC	GTP	(57)
	MtDsbA	888481	Rv2969c	4K6X/4IHU	2013/2012	1.97/1.90	19	2.8, 123	CPAC	ATP	(15,55)
	CdMdbA	2649256	DIP1880	5C00	2015	1.77	16	2.4, 128	CPHC	SSP	(58)

SUPPLEMENTARY FIG. S1. Taxonomy common tree of bacteria encoding DsbA prototypes and summary of DsbA structures deposited in the PDB. Seventeen bacterial species encoding the 22 DsbA enzymes analyzed in this study were selected from the NCBI taxonomy database to generate a common tree of this set of taxa. The obtained phylip tree was visualized and edited in FigTree v1.4 with nodes collapsed at the taxonomic unit of ''Class'' (*node labels*). Bacterial plyla are also shown. F and A denote Firmicutes and Actinobacteria (tetrabacteria group), respectively.

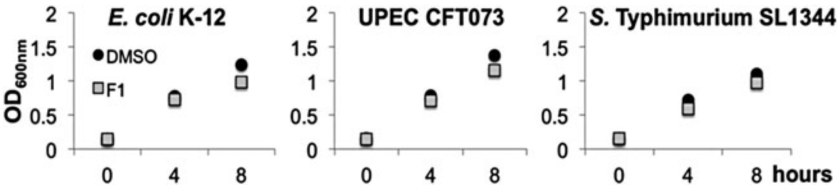

SUPPLEMENTARY FIG. S2. Bacterial growth in the presence of small-molecule EcDsbA inhibitor F1. Wild-type bacterial strains *E. coli* K-12, UPEC CFT073, and *S.* Typhimurium SL1344 were inoculated in liquid lysogeny broth media supplemented with 1 m*M* F1 inhibitor or 0.1% DMSO (carrier control), and culture optical density at 600 nm (OD_{600nm}) was measured at 0, 4, and 8 h of shaking culture at 37°C. Mean ± standard deviation OD_{600} values of $n = 3$ independent replicates are plotted. EcDsbA, *Escherichia coli* DsbA; DMSO, dimethyl sulfoxide; UPEC, uropathogenic *Escherichia coli*.

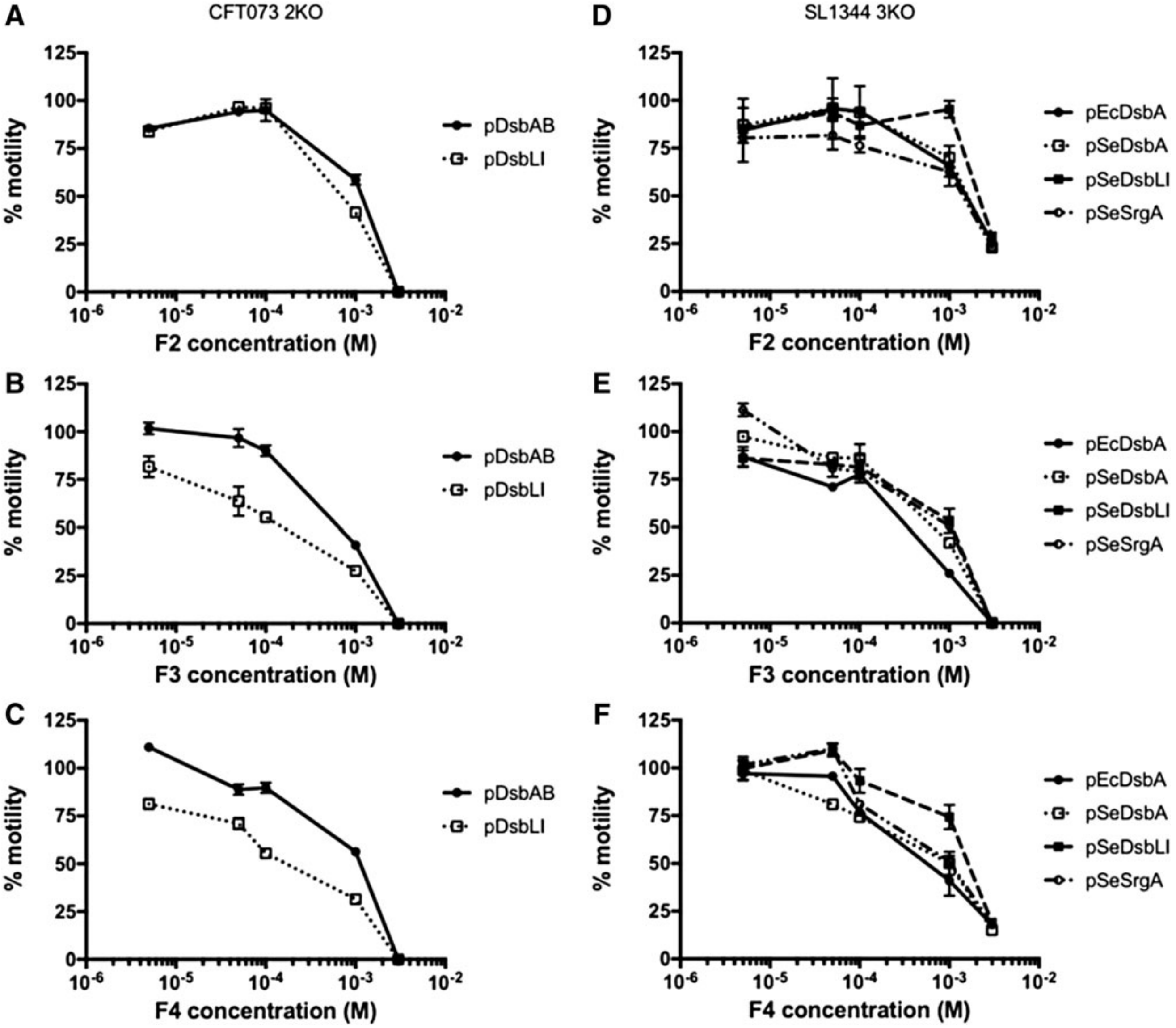

SUPPLEMENTARY FIG. S3. Dose-dependent inhibition of UPEC (A–C) and *S.* Typhimurium (D–F) motility by inhibitors F2, F3, and F4. Dose-response plots of relative motility for **(A–C)** UPEC CFT073 2KO transiently expressing DsbAB (*closed circles*) or DsbLI (*open squares*) and **(D–F)** *S.* Typhimurium SL1344 3KO complemented with EcDsbA (*closed circles*), SeDsbA (*open squares*), SeDsbLI (*closed squares*), or SeSrgA (*open circles*). Relative% motility was calculated by measuring the diameter of the motility zone for each strain in media containing 0.005, 0.05, 0.1, 1, or 3 m*M* of inhibitor F2, F3, or F4 and dividing by the diameter of the same strain swimming in DMSO-containing media (carrier control). Dot plots represent mean relative motility ± SEM of four independent replicates. SeDsbA, DsbA from *S. enterica* serovar Typhimurium; SEM, standard error of the mean.

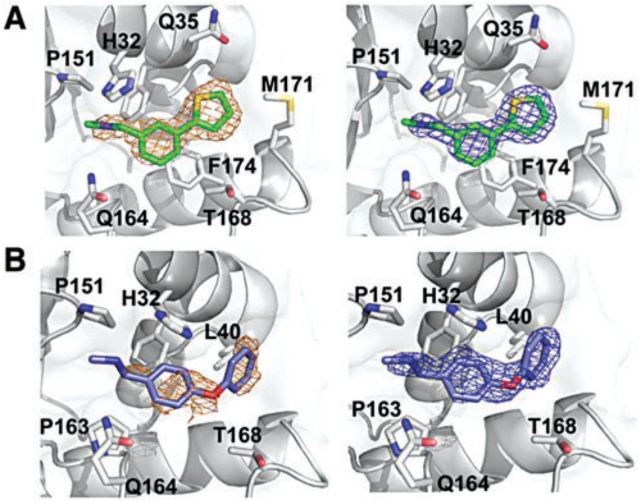

SUPPLEMENTARY FIG. S4. F1 and F3 compounds in complex with EcDsbA. Simulated annealing omit σ_A-weighted mFo-DFc electron density map of compounds F1 (**A**, *left panel*) and F3 (**B**, *left panel*) are contoured at 2.5σ and displayed in *orange* mesh. σ_A-weighted 2mFo-DFc electron density map of compounds F1 (**A**, *right panel*) and F3 (**B**, *right panel*) are contoured at 1σ and displayed in *blue* mesh.

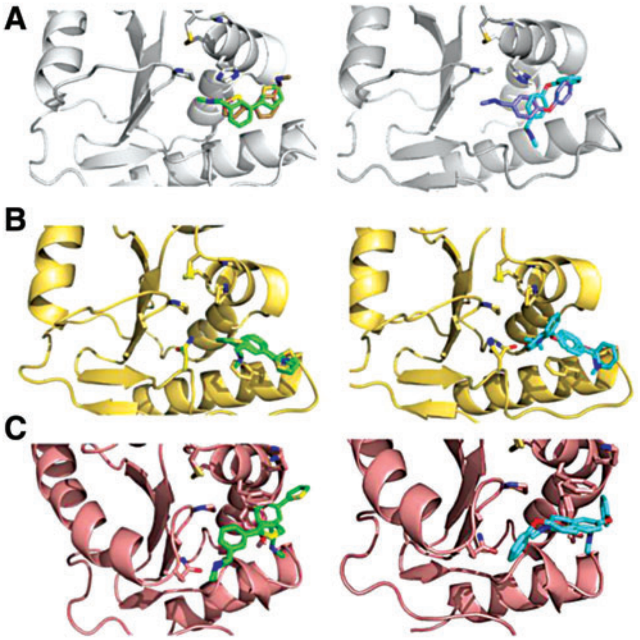

SUPPLEMENTARY FIG. S5. Molecular docking of F1 and F3 inhibitors with EcDsbA, SeSrgA, and SeDsbL. **(A)** Comparison of the crystal structures of EcDsbA-F1 (*light green*, *left panel*) and EcDsbA-F3 (*slate*, *right panel*) with the molecular docking of F1 (*sand*) and F3 (*cyan*) with EcDsbA. **(B, C)** Binding mode of *top* ranked docked poses into SrgA and DsbL groove. **(B)** SrgA with the 3 *top* docked conformations of F1 (predicted affinities range between −5.6 and −5 Kcal/mol, *left panel*) and F3 (predicted affinities range between −5.7 and −5.6 Kcal/mol, *right panel*). **(C)** DsbL with the three *top* docked conformations of F1 (predicted affinities range between −5.8 and −5.5 Kcal/mol, *left panel*) and F3 (predicted affinities range between −5.3 and −5.1 Kcal/mol, *right panel*).

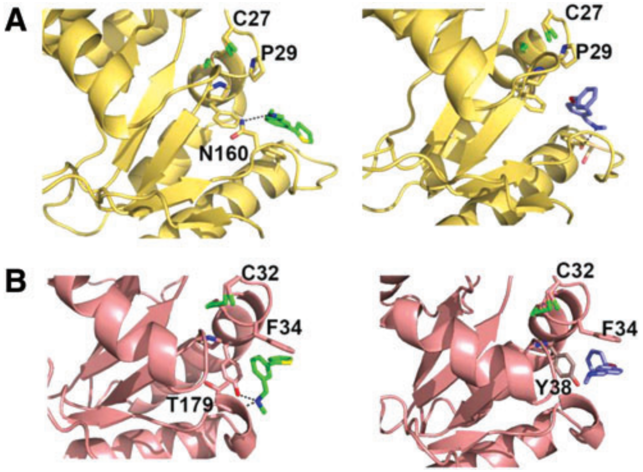

SUPPLEMENTARY FIG. S6. Docking of F1 and F3 with SrgA and DsbL showing the binding mode of *top* ranked docked poses of F1 (*green*) and F3 (*slate blue*) into SrgA **(A)** and DsbL **(B)** hydrophobic clefts. All four structures are rotated 90° around the vertical axis with respect to the orientation shown in Figure 7D, E and show that no steric clashes were observed between the compounds and the residues in the DsbA homologues.

Supplementary Table S1. X-ray Crystallography Data Collection and Refinement Statistics

Data collection	*Compound 1 6BR4*	*Compound 3 6BQX*
Space group	*C*2	*C*2
Cell dimensions (Å) (a, b, c)	(116.22, 63.94, 74.51)	(116.17, 64.01, 74.10)
Angles	$\alpha=\gamma=90°$ $\beta=126.15°$	$\alpha=\gamma=90°$ $\beta=125.73°$
Resolution (Å)[a]	52.8–1.99 (2.06–1.99)	48.4–1.99 (2.06–1.99)
Total number of observations	1,09,444	1,10,612
Number of unique observations	29,804	30,096
Multiplicity	3.7 (3.4)	3.7 (3.4)
Data completeness (%)	98.0 (94.1)	98.9 (95.8)
$\langle I/\sigma I \rangle$	16.8 (3.5)	13.7 (2.9)
R_{merge}(%)[b]	4.7 (26.1)	5.3 (33.1)
Refinement		
Resolution (Å)	52.8–1.99 (2.02–1.99)	30.3–1.99 (2.04–1.99)
No. of reflections	29,800	27,019
R_{work}[c]/R_{free}[d]	0.17/0.21 (0.46/0.53)	0.18/0.24 (0.24/0.31)
No. atoms: protein	2972	2951
No. atoms: water	291	236
Wilson B	34.45	33.30
B factor ($Å^2$)–all	35.86	44.77
B factor ($Å^2$)–water	42.13	46.92
B factor ($Å^2$)–protein	35.24	44.41
R.m.s. deviations		
Bond lengths (Å)	0.007	0.010
Bond angles (°)	0.919	1.027
Ramachandran plot		
Residues in most favored/additionally allowed regions (%)	98.12/1.88	97.3/2.7
MolProbity score (percentile)	1.08 (100th)	1.37

[a]Values in parentheses refer to the highest resolution shell.
[b]Agreement between intensities of repeated measurements of the same reflections can be defined as:

$$R_{merge} = \frac{\sum_{hkl}\sum_{i=1}^{n}|I_i(hkl) - \bar{I}(hkl)|}{\sum_{hkl}\sum_{i=1}^{n}I_i(hkl)}$$

where $I_i\,(hkl)$ are individual values and $\bar{I}(hkl)$ is the mean value of the intensity of reflection *hkl*.
[c]$R_{fac}=\sum_h |F_o - F_c|/\sum_h|F_o|$, where F_o and F_c are the observed and calculated structure-factor amplitudes for each reflection "h."
[d]R_{free} was calculated with 5% of the diffraction data selected randomly and excluded from refinement.

Supplementary Table S2. Table of Bacterial Strains and Plasmids

Strain name	*Description*	*Ref.*
MG1655	*E. coli* K-12 (OR:H48:K-)	(8)
CFT073	UPEC isolate (O6:K2:H1)	(51)
CFT073 2KO	CFT073*dsbABdsbLI*	(68)
CFT073 2KO + pDsbAB	CFT073*dsbABdsbLI* pDsbAB	(68)
CFT073 2KO + pDsbLI	CFT073*dsbABdsbLI* pDsbLI	(68)
CFT073 2KO + empty vector	CFT073*dsbABdsbLI* pUC19	(68)
SL1344	*Salmonella enterica* serovar Typhimurium	(31)
SL1344 3KO	SL1344 *dsbA,dsbLI,srgA*	(30)
SL1344 3KO + pDsbA	SL1344 *dsbA,dsbLI,srgA* pSeDsbA	(30)
SL1344 3KO + pDsbLI	SL1344 *dsbA,dsbLI,srgA* pSeDsbLI	(30)
SL1344 3KO + pSrgA	SL1344 *dsbA,dsbLI,srgA* pSeSrgA	(30)
SL1344 3KO + pEcDsbA	SL1344 *dsbA,dsbLI,srgA* pEcDsbA	This study
SL1344 3KO + empty vector	SL1344 *dsbA,dsbLI,srgA* pWSK29	(30)

UPEC, uropathogenic *Escherichia coli.*
